# High flow nasal cannula and low level continuous positive airway pressure have different physiological effects during de novo acute hypoxemic respiratory failure

**DOI:** 10.1186/s13613-024-01408-w

**Published:** 2024-11-23

**Authors:** Samuel Tuffet, Mohamed Ahmed Boujelben, Anne-Fleur Haudebourg, Tommaso Maraffi, François Perier, Pascale Labedade, Elsa Moncomble, Ségolène Gendreau, Matthieu Lacheny, Emmanuel Vivier, Armand Mekontso-Dessap, Guillaume Carteaux

**Affiliations:** 1 Hôpital Saint Joseph Saint Luc, Médecine Intensive Réanimation, 20 quai Claude Bernard, Lyon, France; 2 Groupe de Recherche Clinique CARMAS, Faculté de Santé, Université Paris Est-Créteil, Créteil Cedex, 94010 France; 3 INSERM U955, Institut Mondor de Recherche Biomédicale, Créteil Cedex, 94010 France; 4 CHU Henri Mondor-Albert Chenevier, Service de Médecine Intensive Réanimation, Assistance Publique- Hôpitaux de Paris, 51, Avenue du Maréchal de Lattre de Tassigny, Créteil Cedex, 94010 France; 5 Service de Médecine Intensive-Réanimation, Centre Hospitalier Intercommunal de Créteil, Créteil, France; 6 Service de réanimation, Centre Hospitalier de La Rochelle, La Rochelle, France

**Keywords:** Acute hypoxemic respiratory failure, High flow nasal cannula, Continuous positive airway pressure, Noninvasive ventilation, Respiratory support

## Abstract

**Background:**

Large tidal volumes during de novo acute hypoxemic respiratory failure (AHRF) may promote patient self-inflicted lung injury. Tidal volume assessment under high flow nasal cannula (HFNC) is not routinely feasible at the bedside. Our objective was to determine whether tidal volume during low-level continuous positive airway pressure (CPAP) could predict tidal volume during HFNC and to compare the physiological effects of HFNC and low-level CPAP.

**Methods:**

Prospective, single-center study including 29 de novo AHRF patients treated with HFNC (50 to 60 L.min^− 1^). Patients were monitored using electrical impedance tomography during HFNC then CPAP at 4 cmH_2_O. Tidal volume during HFNC was calculated based on tidal impedance variation. The ability of tidal volume under low-level CPAP to predict tidal volume under HFNC was explored using Bland-Altman analysis. CPAP and HFNC were compared in terms of tidal volume, minute ventilation, respiratory comfort, dyspnea, oxygenation, ventilation distribution, end-expiratory lung volume, thoraco-abdominal asynchrony and recruitment.

**Results:**

Under HFNC, patients had a tidal volume of 6.6 (5.9–8.7) mL.kg^− 1^ PBW. 20 (69%) patients exhibited a tidal volume between 4 and 8 mL.kg^− 1^ PBW, while in 5 (17%) patients it exceeded 9 mL.kg^− 1^ PBW. Tidal volume under CPAP was higher (9.4 (8.3–11) mL.kg^− 1^ PBW, *p* < 0.001). Tidal volumes under CPAP and under HFNC were modestly correlated (Spearman *r* = 0.50, *p* = 0.005). Bland-Altman analysis showed a bias of 2.4 mL.kg^− 1^, with limits of agreement ranging from − 1.1 mL.kg^− 1^to 5.9 mL.kg^− 1^. Nevertheless, a larger (> 11.5 mL.kg^− 1^ PBW ) tidal volume under low-level CPAP predicted a larger (> 9 mL.kg^− 1^ PBW ) tidal volume under HFNC with 80% sensitivity and 96% specificity. Low-level CPAP was associated with increased minute ventilation, end-expiratory lung volume, and oxygenation as compared to HFNC. It decreased signs of respiratory distress in the most severe patients but was associated with lower comfort compared to HFNC.

**Conclusion:**

Among ICU patients with de novo AHRF, tidal volume under HFNC was mostly protective. Tidal volume during CPAP at 4 cmH_2_O did not predict tidal volume during HFNC. Such low-level CPAP was associated with increased tidal volume, minute ventilation, end-expiratory volume, and oxygenation.

**Trial registration:**

ClinicalTrials.gov ID NCT03919331. Registration date: 2019-03-26.

**Supplementary Information:**

The online version contains supplementary material available at 10.1186/s13613-024-01408-w.

## Introduction

During de novo acute hypoxemic respiratory failure (AHRF), large tidal volumes are associated with poor outcome in patients under noninvasive ventilation (NIV) [1, 2], raising concern about the potential involvement of patient self-inflicted lung injury (P-SILI) [3, 4]. Several devices are available to provide oxygen to such patients, with high flow oxygen through nasal cannula (HFNC) being widely adopted in intensive care units due to its clinical benefits compared to NIV and conventional oxygen therapy [5–8]. However, assessing tidal volume under HFNC is not routinely feasible at the bedside and data are scarce in the literature, leaving the prognostic impact of tidal volume under HFNC during de novo AHRF unknown.

HFNC has been shown to produce several physiological effects compared to conventional oxygen therapy [9], including an increase in end-expiratory lung volume due to the generation of positive airway pressure [6, 9]: with a flow rate of 50–60 L.min^− 1^, HFNC can generate a positive airway pressure approximating 4 cmH_2_O [10–12].

We hypothesized that Continuous Positive Airway Pressure (CPAP) set at a similarly low level of positive airway pressure to that generated by HFNC would result in comparable tidal volumes and could therefore be used to predict HFNC’s effect on tidal volume in clinical practice. Our primary objective was to compare the tidal volume during a low level of CPAP with that measured during HFNC in patients with de novo AHRF.

Additionally, CPAP has regained interest due to its frugality [13], making it suitable for use outside the ICU or constrained conditions [14–17]. Notably, most CPAP devices available for use outside the ICU provide a low level of CPAP [15, 17]. Physiological effects of such low-level CPAP have not been compared to those of HFNC. Therefore, our secondary objective was to compare the effects of low-level CPAP and HFNC on end-expiratory lung volume, oxygenation, distribution of ventilation, and thoraco-abdominal asynchrony.

## Methods

This was a prospective, single-center, physiological study conducted in the medical intensive care unit of Henri Mondor Hospital, Créteil, France. The study was approved by the ethics committee “Comité de Protection des Personnes Ile de France 4” (2018-A02972-53). This research had been registered on clinicaltrial.gov (NCT03919331). A detailed description of the methods is provided in the online supplementary material.

### Patients

Adult patients, hospitalized for de novo acute hypoxemic respiratory failure (defined as P_a_O_2_/F_i_O_2_ < 300 mmHg and P_a_CO_2_ ≤ 45 mmHg), with a respiratory rate greater than 25.min^-1^, and placed under HFNC as part of standard care, were eligible. Non-inclusion criteria were systolic arterial pressure < 90 mmHg, mean arterial pressure < 65 mm Hg, use of vasopressors, impaired consciousness (Glasgow coma score ≤ 12), contraindication to CPAP (e.g., facial trauma), contraindication to electrical impedance tomography (EIT), AHRF explained solely by cardiogenic pulmonary edema, asthma or chronic obstructive pulmonary disease exacerbation.

### Protocol

The study consisted of three steps:

1. Initial HFNC step (HFNC 1): A 10-minute period during which F_i_O_2_ and gas flow were left as set by the clinician.
2. A CPAP step: A 10-minute period during which patients were placed under CPAP using an ICU ventilator via an oronasal mask. A heat and moisture exchanger was inserted in the ventilator circuit. The CPAP level was set at 4 cmH_2_O, and F_i_O_2_ was kept unchanged. Absence of significant leakage was verified by comparing expired and insufflated volumes.
3. Second HFNC step (HFNC 2): A 10-minute period with the same F_i_O_2_ and flow rate as HFNC 1.

All measurements were done during the last minute of each step.

### Collected data

#### General data

During each step, blood pressure, heart rate, respiratory rate (RR), and transcutaneous oxygen saturation (SpO_2_) were collected. Arterial blood gases were taken at the end of the HFNC 1 and CPAP steps if an arterial catheter was present. Otherwise, a single arterial blood gas was collected at the end of HFNC 1. Changes in discomfort and dyspnea between HFNC 1 and CPAP were assessed using a 5-point Likert scale ranging from − 2 (much less discomfort or dyspnea) to + 2 (much more discomfort or dyspnea).

#### Electrical impedance tomography (EIT)

Patients were monitored using the Enlight 1800 EIT device (TIMPEL SA, São Paulo, Brazil). Two hemi-belts applied to the patient’s thorax collected thoracic impedance variations, that are proportional to intrathoracic aeration variations. During the HFNC phases, only electrical impedance was collected. During the CPAP phase, a pneumotachograph was inserted on the ventilator circuit to record tidal volume.

#### Thoraco-abdominal asynchrony

Thoracic and abdominal respiratory movements were recorded using two stretch-sensitive respiratory transducers connected to a wireless transmitter (BioNomadix^®^ Respiration Transducer, Biopac systems, Goleta, CA, USA). The signals were then processed using AcqKnowledge version 4.3 (Biopac systems, Goleta, CA, USA).

### Computed data

#### Tidal volume estimation under HFNC

The tidal volume under HFNC was calculated from the tidal impedance changes. The proportionality ratio between volume changes and electrical impedance changes was calculated during the CPAP step and used to convert tidal impedance changes during HFNC into tidal volumes [9]. Corrected minute ventilation was computed as minute ventilation multiplied by the ratio of the patient’s PaCO_2_ to 40 mm Hg [9].

#### Pendelluft

Pendelluft was calculated using a previously published method [18, 19]. A detailed description of this method is available in the supplement.

#### End-expiratory lung volume

Changes in end-expiratory lung volume (EELV) between steps were calculated from the changes in end-expiratory lung impedance (EELI). EELI was first averaged over the last minute of each step. Then the proportionality ratio between changes in volume and changes in electrical impedance was applied. EELI during HFNC 1 was taken as the reference.

#### Ventilation maps

Ventilation maps representing the distribution of ventilation at the pixel level were obtained for each condition using dedicated software (TIMPEL SA, São Paulo, Brazil). Each map was composed of 1024 pixels. To account for potential tidal volume variations between conditions, ventilated pixels were defined as follows [20, 21]: for each map, the volume received by each pixel was calculated by multiplying the tidal volume by the fraction of ventilation received by that pixel. The pixel receiving the most volume (Pixel_max_) was identified for each map. Among the three Pixel_max_ obtained in a given patient (HFNC1, CPAP, HFNC2), the one receiving the lowest volume was taken as reference (Pixel_REF_). A pixel was considered ventilated if it received a volume greater than 10% of the volume received by the Pixel_REF_. A pixel was considered to represent potentially aerated lung if it was ventilated in at least one condition.

From the ventilation maps were calculated.

- The number of ventilated pixels (Pixel_VENT_), estimating the functional size of the lung. The changes in functional lung size between the three conditions were then calculated in absolute change (Δ Pixel_VENT, ABS_) and relative value (Δ Pixel_VENT, REL_ = Δ Pixel_VENT, ABS_ / Pixel_VENT_).
- The global inhomogeneity index (GI) [22].
- The distribution of ventilation (anterior versus posterior), expressed as a percentage of total ventilation.

#### Thoraco-abdominal asynchrony

The thoraco-abdominal phase angle, an objective measure of thoraco-abdominal asynchrony, was calculated [23–25]. Detailed description of the calculation is provided in the supplementary material.

### Statistics

Continuous variables are expressed as median (interquartile range); categorical variables are expressed as number (percentage). Differences between the three conditions were examined using a Friedman test, followed by Wilcoxon paired tests with Benjamini-Hochberg correction. The relationship between tidal volume under HFNC and CPAP was assessed using Spearman correlation and Bland-Altman analysis. The performance of a larger tidal volume under CPAP in diagnosing a larger tidal volume under HFNC (i.e., > 9 mL.kg^− 1^ PBW a threshold associated with intubation or death in patients under NIV [26]) was assessed by ROC curve analysis (to determine the best cut-off for CPAP tidal volume), sensitivity and specificity. Patients were separated into two groups according to the median ROX index [27] at inclusion to determine the differential effects of CPAP in the most severe patients. Statistics were performed using GraphPad Prism software version 9.4.1 (GraphPad Software, LLC).

### Ethics

Patients were included after receiving oral and written information and signing a consent form.

## Results

### Patients

Thirty patients were assessed. Data from the first patient were excluded due to unstable EIT recordings. Characteristics of the 29 analyzed patients, included 15 (11–19) hours after starting HFNC, are provided in Table 1.

**Table 1:** Patient’s characteristics

Variables	All patients (*n* = 29)
Age (years)	63 (54–69)
Male sex – n (%)	23 (79)
BMI (kg.m^− 2^)	24 (20–27)
SAPS 2 at admission	39 (27–48)
Current or past smoking – n (%)	10 (34)
Reason for de novo acute respiratory failure – n (%)	
Community-acquired pneumonia	18 (62)
Including COVID-19	7 (24)
Including pneumonia related to immunosuppression	5 (17)
Hospital-acquired pneumonia	5 (17)
Including pneumonia related to immunosuppression	3 (10)
Aspiration – n (%)	1 (3)
Other – n (%)	5 (17)
Bilateral pulmonary infiltrates – n (%)	20 (69)
Respiratory rate (breaths.min^− 1^)	24 (19–28)
Heart rate (beat.min^− 1^)	87 (72–97)
Systolic arterial pressure (mm Hg)	123 (117–139)
Diastolic arterial pressure (mm Hg)	69 (57–75)
HFNC data	
Flow (L.min^− 1^)	50 (50–60)
F_i_O_2_ (%)	70 (50–100)
Arterial Blood Gas	
pH	7,46 (7,43 − 7,49)
[HCO_3_^−^] (mmol.L^− 1^)	24,4 (22,1–26,9)
P_a_CO_2_ (mm Hg)	33 (30,5–37)
P_a_O_2_ (mm Hg)	78 (68–109)
P_a_O_2_/F_i_O_2_ (mm Hg)	123 (101–169)
S_a_O_2_ (%)	95 (93–98)
Lactate (mmol.L^− 1^)	1.2 (0.8–1.9)
Ventilatory ratio	1.4 (1.12–1.8)

BMI: Body Mass Index; SAPS 2: Simplified Acute Physiology Score 2 (Le Gall JR, Lemeshow S, Saulnier F. A new Simplified Acute Physiology Score (SAPS II) based on a European/North American multicenter study. JAMA. 1993 Dec 22–29;270 [24]:2957-63.); HFNC: High Flow Nasal Cannula; S_p_O_2_: oxygen saturation by pulse oximeter; F_i_O_2_: inspired fraction of oxygen; P_a_CO_2_: arterial carbon dioxide pressure; P_a_O_2_: arterial oxygen pressure; S_a_O_2_: arterial oxygen saturation

### Tidal volume and minute ventilation

**Fig. 1:**
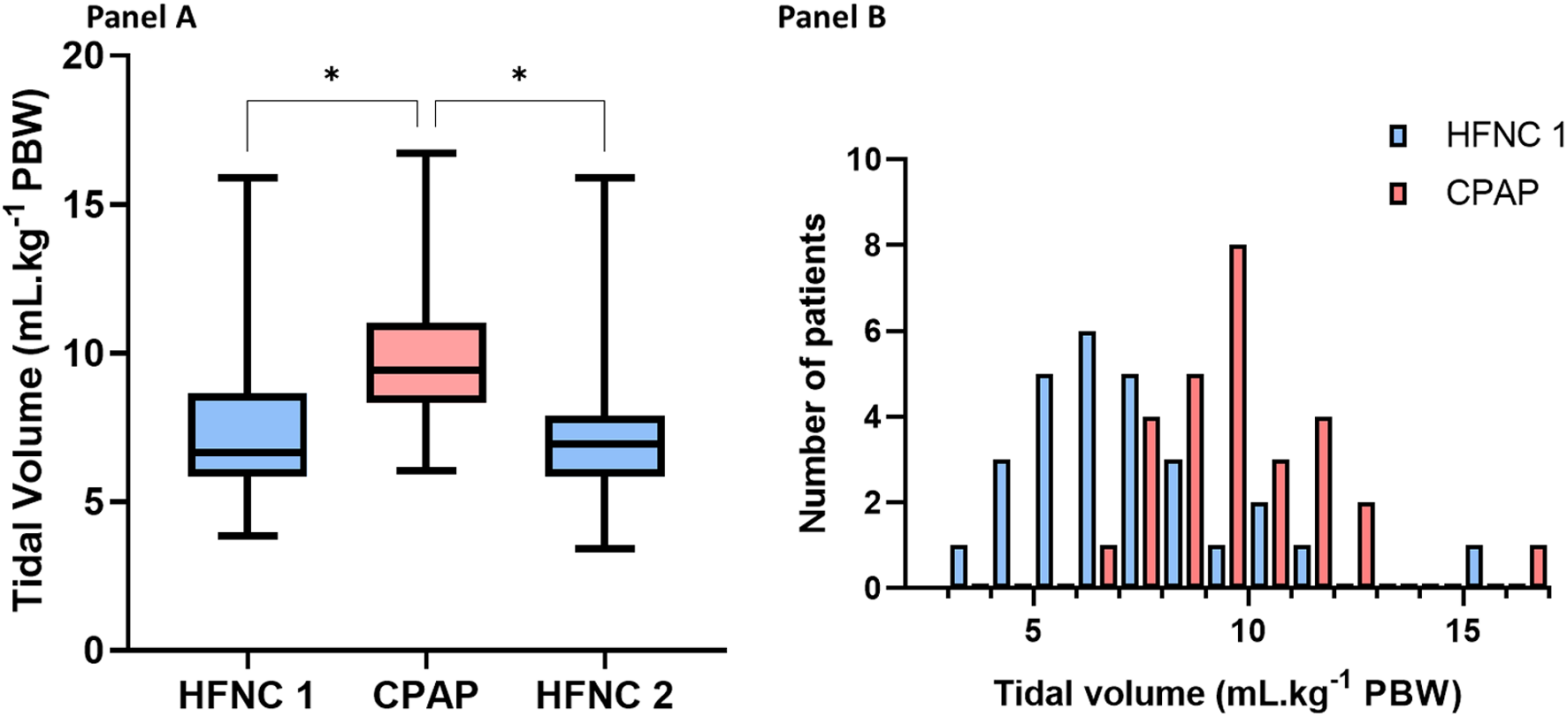
Comparison of tidal volumes between HFNC and CPAP. Legend: Panel **A**. Statistical comparison of tidal volume between HFNC 1, CPAP and HFNC 2. * denotes *p* < 0.01. Panel **B**. Distribution of tidal volumes among 29 patients under HFNC 1 (blue) and CPAP (red). HFNC: High Flow Nasal Cannula; CPAP: Continuous Positive Airway Pressure; PBW: predicted body weight

Ventilatory data during the HFNC 1 and CPAP are summarized in Table 2. Data comparing the three conditions are available in Table S1. Under HFNC 1 (at baseline), patients had a tidal volume of 6.6 (5.9–8.7) mL.kg^− 1^ PBW. 20 (69%) patients exhibited a tidal volume between 4 and 8 mL.kg^− 1^ PBW while the tidal volume exceeded 9 mL.kg^− 1^ PBW in 5 (17%) patients (Fig. 1).

**Table 2:** Comparison between HFNC 1 and CPAP

Variable	HFNC 1	CPAP	Adjusted *p*-value
F_i_O_2_ (%)	70 (50–100)	70 (50–100)	NA
Flow (L.min^− 1^)	50 (50–60)	NA	NA
Respiratory rate (.min^− 1^)	24 (19–28)	24 (20–30)	0.09
Tidal volume (mL)	453 (353–578)	629 (551–686)	< 0.001
Tidal volume/PBW (mL.kg^− 1^ PBW)	6.6 (5.9–8.7)	9.4 (8.3–11)	< 0.001
Minute ventilation (L.min^− 1^)	10.6 (7.9–13.8)	15.8 (12.5–17.5)	< 0.001
Tidal impedance variation _pixel−level_ (arbitrary units)	37.9 (29.3–50.6)	46.9 (38.9–65.7)	< 0.001
Pendelluft (%)	12.1 (8.3–18.5)	9.8 (6.3–14.1)	0.002
EELV variation (mL)	0	170 (43–399)	< 0.001
EELV variation/PBW (mL.kg^− 1^ PBW)	0	2.5 (0.6–5.4)	< 0.001
Anterior EELV variation/PBW (mL.kg^− 1^ PBW)	0	1.1 (0.2–3.1)	< 0.001
Posterior EELV variation/PBW (mL.kg^− 1^ PBW)	0	1.1 (0.1–2.7)	< 0.001
Thoraco-abdominal phase angle (°) (*N* = 20)	15 (9–34)	10 (7–21)	0.51
S_p_O_2_ (%)	96 (95–99)	99 (97–100)	< 0.001
Dyspnea (*n* = 23)	0	0 (-1-1)	0.15
Discomfort (*n* = 23)	0	1 (0–1)	0.02
ROX	5.7 (4.2–8.2)	5.5 (4.1–8.5)	0.55
Anterior distribution (%)	46 (41–54)	41 (37–50)	0.01
Global inhomogeneity index	0.56 (0.53–0.60)	0.58 (0.53–0.60)	0.78
Ventilated pixels (n)	327 (292–356)	358 (341–394)	< 0.001
**Hemodynamic parameters**			
Heart rate (beat.min^− 1^)	87 (72–97)	87 (71–103)	0.09
SAP (mm Hg)	123 (117–139)	134 (115–146)	< 0.01
DAP (mm Hg)	69 (57–75)	74 (61–78)	< 0.01
PP (mm Hg)	55 (49–68)	58 (48–77)	0.28
**Arterial blood gases (** ***n*** ** = 12)**			
P_a_O_2_/F_i_O_2_ (mm Hg)	109 (77–160)	153 (109–194)	< 0.01
P_a_CO_2_ (mm Hg)	33 (31–35)	35 (34–37)	< 0.001
pH	7.48 (7.44–7.5)	7.45 (7.41–7.48)	< 0.001
[HCO_3_^−^] (mmol.L^− 1^)	24.6 (24-26.5)	25.6 (23.6–26.8)	0.11
Corrected minute ventilation (L.min^− 1^)	8.5 (7.4–11.3)	14.6 (11.4–15.8)	< 0.001
Ventilatory ratio	1.39 (1.11–1.67)	2.15 (1.91–2.6)	0.001

Adjusted p-values were computed by the mean of Wilcoxon paired tests with Benjamini-Hochberg correction

Tidal impedance variation _pixel−level_ refers to the sum of the tidal impedance variations of every pixel, with the breath cycle defined for each pixel, thus considering the Pendelluft. End-expiratory lung volume during CPAP is expressed as difference from HFNC1. The change in discomfort and dyspnea between HFNC 1 and CPAP was assessed using a 5-point Likert scale ranging from − 2 (much less discomfort or dyspnea to + 2: much more discomfort or dyspnea)

HFNC: High Flow Nasal Cannula; CPAP: Continuous Positive Airway Pressure; F_i_O_2_: inspired fraction of oxygen; PBW: predicted body weight; EELV: end-expiratory lung volume; S_p_O_2_: oxygen saturation by pulse oximeter; SAP: systolic arterial pressure; DAP: diastolic arterial pressure; PP: pulse pressure; P_a_O_2_: arterial oxygen pressure; F_i_O_2_: inspired fraction of oxygen; P_a_CO_2_: arterial carbon dioxide pressure

**Fig. 2:**
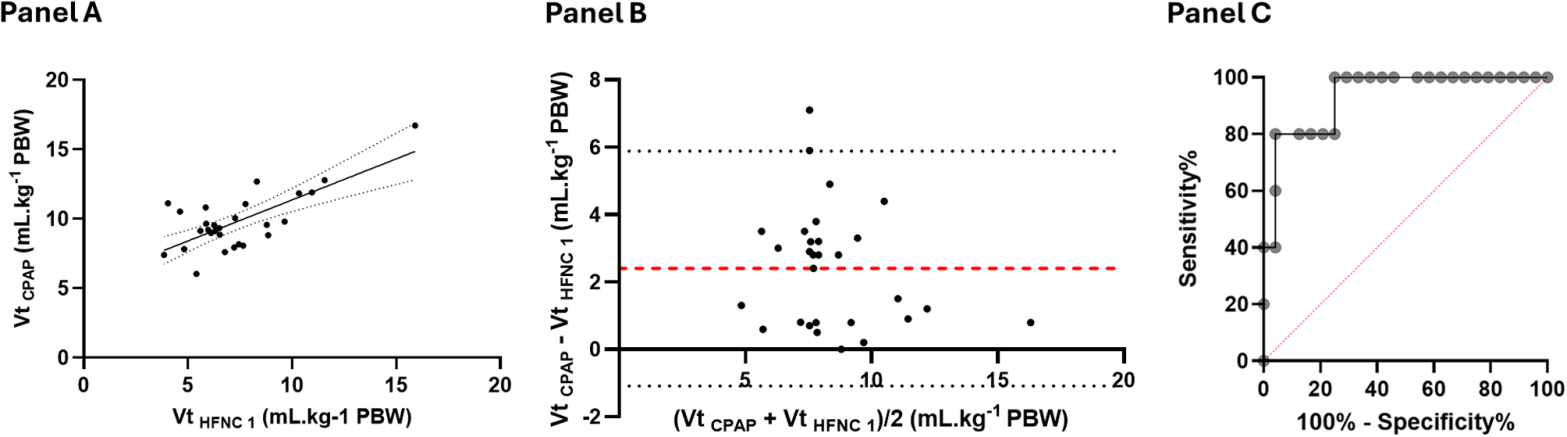
Accuracy of tidal volume during low-level CPAP to predict tidal volume during HFNC. Legend: Panel **A**. Correlation between tidal volume under HFNC and tidal volume under CPAP. Panel **B**. Bland-Altman graph comparing tidal volume under HFNC and tidal volume under CPAP. Panel **C**. ROC curve of tidal volume during CPAP to diagnose tidal volume under HFNC > 9mL.kg-1 PBW. HFNC: High Flow Nasal Cannula; CPAP: Continuous Positive Airway Pressure; PBW: predicted body weight. Vt: tidal volume

Tidal volume under CPAP was significantly higher compared to HFNC (Table 2; Fig. 1). The median tidal volume increase between HFNC 1 and CPAP was 2.8 (0.8–3.4) mL.kg^− 1^ PBW (Figure S2). Tidal volume under HFNC 1 and CPAP were modestly correlated (Spearman *r* = 0.50, *p* = 0.005) (Fig. 2A). Bland-Altman analysis showed a bias of 2.4 mL.kg^− 1^, with limits of agreement ranging from − 1.1 to 5.9 mL.kg^− 1^ PBW (Fig. 2B). The change in tidal volume between HFNC1 and CPAP was inversely correlated to the tidal volume during HFNC, with patients with higher tidal volume having the smallest change or no change at all (Figure S3). Regarding intra-tidal gas redistribution, CPAP was associated with significantly less Pendelluft than HFNC 1: 9.8% (6.3–14.1) vs. 12.1% (8.3–18.5), *p* = 0.002.

The area under the ROC curve for a tidal volume under CPAP to predict a larger (> 9 mL.kg^− 1^ PBW) tidal volume under HFNC was 0.93 (95% CI: 0.83 to 1) (Fig. 2). A tidal volume > 11.5 mL.kg^− 1^ PBW under CPAP predicted a tidal volume > 9mL.kg^− 1^ PBW under HFNC with 80% (95% CI: 38 to 99) sensitivity and 96% (95% CI: 80 to 100) specificity. 11 (38%) patients were eventually intubated. Their tidal volumes were not significantly higher than those of non-intubated patients (HFNC 1: 7.2 mL.kg^− 1^ PBW [5.2–8.1] vs. 6.1 mL.kg^− 1^ PBW [6.3–8.8], *p* = 0.17; CPAP: 9.3 mL.kg^− 1^ PBW [7.9–9.8] vs. 9.5 mL.kg^− 1^ PBW [8.7–11.1], *p* = 0.72). A tidal volume > 9 mL.kg-1 PBW during HFNC or > 11.5 mL.kg-1 PBW during CPAP was not associated with intubation, as determined by Fisher exact test (*P* > 0.99 for both comparisons) (Table S2).

Minute ventilation was significantly higher with CPAP compared to HFNC.

### Gas exchange

At the same FiO_2_, CPAP resulted in higher SpO_2_ (Table 2). Among the 12 patients with blood gases measured under CPAP, the PaO_2_/FiO_2_ ratio significantly increased (Table 2). Changes in PaO_2_/FiO_2_ ratio between HFNC 1 and CPAP did not correlate with changes in EELV, tidal volume, or tidal volume distribution.

Despite increased tidal volume and minute ventilation, CPAP was associated with higher PaCO_2_ [35 mmHg (34-37) for CPAP vs. 33 mmHg (31-35) for HFNC 1, *p* < 0.01], suggesting increased dead space. As a result, corrected minute ventilation and ventilatory ratio were higher during CPAP. Detailed data from the 12 patients with CPAP blood gases are presented in Table S3.

### End-expiratory lung volume, recruitment and ventilation distribution

EELV under CPAP was significantly higher than under HFNC (median 2.5 mL.kg^− 1^ PBW vs. 0 for CPAP and HFNC respectively, *p* < 0.001) (Table 2). The increase in EELV was similarly distributed between anterior and posterior regions (*p* = 0.77 for anterior variation vs. posterior variation). CPAP increased functional lung size, estimated by the number of ventilated pixels (median 358 vs. 327, *p* < 0.001) (Table 2). The relative increase in ventilated pixels between HFNC 1 and CPAP was 13% (7-20). Ventilation distribution slightly favored posterior areas under both supports, with CPAP resulting in more posterior ventilation (median anterior distribution of ventilation 41% vs. 46% for CPAP and HFNC respectively, *p* = 0.01) (Table 2). Ventilatory inhomogeneity, measured by the global inhomogeneity index, did not significantly differ between HFNC and CPAP (*p* = 0.78).

### Thoraco-abdominal phase angle, discomfort and dyspnea

Thoraco-abdominal phase angle was evaluated in 20 patients (not performed in 7 COVID-19 patients and 2 patients with highly resistant bacteria due to potential transmission concerns). The phase angle was similar between CPAP and HFNC (*p* = 0.51) (Table 2). In patients with greater respiratory distress (phase angle greater than the median value), CPAP was associated with a significant decrease in thoraco-abdominal phase angle [10.6° (5.6; 20) for CPAP versus 33.5° (24.4–42.7) for HFNC 1, *p* = 0.03].

Although CPAP was associated with more discomfort (*p* = 0.02), it did not increase dyspnea (*p* = 0.15) (Table 2).

The results of the analysis according to ROX score at inclusion is available in the supplement (Table S4).

## Discussion

The main results of our study are as follows: (i) tidal volume under HFNC was mostly in a protective range in our series; (ii) tidal volume under low-level CPAP does not accurately predict tidal volume under HFNC; (iii) low-level CPAP has different physiological effects than HFNC when used in patients during de novo AHRF. The original features of our study include the conversion of electrical impedance data into absolute volumes specifically during HFNC. While others have made similar conversions in different settings, this is the first application with HFNC. We also provided a comprehensive physiological comparison of HFNC and low-level CPAP.

### Tidal volume under HFNC

We report the largest cohort of patients under HFNC for AHRF with absolute values of tidal volume. Large tidal volumes could represent a significant contributor of P-SILI [3, 26]. In our series, most patients exhibited protective tidal volumes, and only 5 (17%) had tidal volumes greater than 9 mL.kg^− 1^ PBW. This finding is consistent with previously published smaller cohorts [28].

We found that tidal volume under low-level CPAP failed to accurately predict tidal volume under HFNC. Despite significant correlation between both tidal volumes, Bland-Altman analysis showed substantial bias and broad limits of agreement. Nevertheless, a larger tidal volume during CPAP (> 11.5 mL.kg^− 1^ PBW) exhibited good sensitivity and specificity for diagnosing a larger high tidal volume under HFNC (> 9 mL.kg^− 1^ PBW). Whether tidal volume has the same prognostic value under HFNC as under NIV in de novo AHRF remains unknown and deserves future studies. In our series, neither tidal volume under HFNC nor CPAP was associated with the need for intubation, consistent with previous observations [29].

### Comparison of the physiological effects of CPAP and HFNC

To our knowledge, this is the first study comparing the detailed physiological effects of HFNC and low-level CPAP in patients with de novo AHRF. Compared to HFNC, low-level CPAP increased tidal volume, minute ventilation, end-expiratory volume, posterior distribution of ventilation, and oxygenation. The rise in EELV and the rise in tidal volume were in the same range. It also decreased signs of respiratory distress in the most severe patients. These physiological effects are important because some CPAP devices, such as certain open valves, which are particularly used in constrained conditions and outside intensive care units [15, 17], typically result in low levels of CPAP.

The increase in tidal volume and minute ventilation under CPAP, despite increased PaCO_2_, suggests an increase in dead space. This can be attributed to: (1) increased instrumental dead space related to the oronasal mask; (2) disappearance of anatomical dead space washout present under HFNC [28, 30, 31]; and (3) possible increase in alveolar dead space under CPAP. The increase in tidal volume varied significantly among patients (0 to 7.1 mL.kg^− 1^ PBW). This suggests that while instrumental dead space and loss of anatomical dead space washout are certain, alveolar dead space may increase in some patients and decrease in others. The negative correlation between the difference in tidal volume from HFNC 1 to CPAP and tidal volume under HFNC 1 suggests that dead space washout’s contribution to carbon dioxide clearance under HFNC is prominent when tidal volume is low.

Higher tidal volumes can result from increased respiratory effort and/or decreased respiratory system impedance. Our data suggest alveolar recruitment with CPAP, evidenced by increased EELV and ventilated pixels, which could increase respiratory system compliance. In our study, the thoraco-abdominal phase angle was not significantly different between CPAP and HFNC. Previous studies [18, 32] indicate similar respiratory effort under CPAP and HFNC, suggesting that increased tidal volume under CPAP is accompanied by increased compliance, allowing higher tidal volumes with similar muscle pressure and driving pressure.

Our study demonstrates that low-level CPAP is associated with higher EELV and recruitment compared to HFNC, suggesting higher end-expiratory pressure. HFNC induces a positive expiratory pressure [9, 28, 33], which can exceed 4 cmH_2_O [34] in healthy volunteers [33, 34] or patients without de novo AHRF [35]. Studies on AHRF patients showed expiratory pressures < 4 cmH_2_O for gas flows used in clinical practice [10], confirming the impact of breathing pattern on airway pressure [36]. Mouth closure’s impact on airway pressure during HFNC is well established [12, 34]. Our study did not instruct patients to keep their mouths closed, reflecting real-life physiological effects of HFNC. The heterogeneity in end-expiratory lung impedance variation may partly relate to whether the mouth was open or closed. The choice of 4 cmH_2_O for low-level CPAP is therefore questionable. Whether lower levels of CPAP may better predict tidal volume under HFNC in the clinical scenario warrants further research.

Low-level CPAP improved oxygenation compared to HFNC, consistent with previous studies [29, 32]. This improvement may result from: (i) alveolar recruitment improving ventilation/perfusion ratios; (ii) redistribution of tidal volume to posterior lung zones reducing low ventilation/perfusion areas; and (iii) potentially lower delivered FiO_2_ during HFNC compared to set FiO_2_, especially in the case of significant respiratory drive and effort [29, 36, 37].

### Limits

Our study has several limitations. First, it is a single-center study with a relatively small number of patients, limiting its power, especially in assessing the prognostic impact of tidal volume. However, our diverse population of de novo AHRF patients, similar tidal volumes under HFNC to other studies [5], and comparable intubation rates to large cohorts [5] tend to make our results generalizable. Second, we did not measure inspiratory effort. The observed increase in EELV, ventilated pixels, dorsal ventilation and improved oxygenation during CPAP could alternatively be explained by increased respiratory effort, leading to intra-tidal recruitment, which may be harmful. Although the findings of decreased Pendelluft and similar levels of dyspnea do not support this explanation, the lack of inspiratory effort measurement precludes definitive conclusions. Third, the physiological effects of prolonged CPAP were not studied. Fourth, we did not standardize mouth closure or opening in order to reflect clinical practice. Fifth, Pendelluft values in our patients were lower than those described elsewhere [18, 19, 38]. This may be due to differences in patient selection and/or discrepancies in pixel-by-pixel impedance data accuracy between the two different EIT devices used. Nevertheless, the interpretation of this parameter’s variation between the two different ventilatory supports remains relevant. Sixth, the volume data from EIT do not cover the entire lung field, possibly missing some localized aeration variations. Seventh, using a pragmatic approach, we included patients who still exhibited hypoxemia and high respiratory rate despite receiving high-flow nasal oxygen. This may constitute an inclusion bias. Finally, the impact of the physiological differences between HFNC and low-level CPAP on patient outcomes cannot be predicted from our study.

## Conclusion

Among ICU patients with de novo AHRF, tidal volume under HFNC was mostly protective. Tidal volume during CPAP 4 cmH_2_O did not predict tidal volume during HFNC 50 to 60 L.min^− 1^. Low-level CPAP was associated with increased tidal volume, minute ventilation, end-expiratory volume, and oxygenation.

## Electronic supplementary material

Below is the link to the electronic supplementary material.

Supplementary Material 1

## Data Availability

The datasets used and/or analyzed during the current study are available from the corresponding author on reasonable request.

## Abbreviations

ARDS: Acute respiratory distress syndrome
BMI: Body Mass Index
CPAP: Continuous Positive Airway Pressure
EELV: End-expiratory lung volume
F_i_O_2_: Inspired fraction of oxygen
GI: Global inhomogeneity index
HFNC: High Flow Nasal Cannula
P_a_CO_2_: Arterial carbon dioxide pressure
P_a_O_2_: Arterial oxygen pressure
PBW: Predicted body weight
RR: Respiratory rate
S_a_O_2_: Arterial oxygen saturation
SAPS 2: Simplified Acute Physiology Score 2
SOFA: Sequential Organ Failure Assessment
S_p_O_2_: Oxygen saturation by pulse oximeter
Vt: Tidal volume
