# Supplementary material for: High flow nasal cannula and low level continuous positive airway pressure have different physiological effects during de novo acute hypoxemic respiratory failure
Source: Ann Intensive Care. 2024 Nov 23;14:171. doi: 10.1186/s13613-024-01408-w (PMC11584821; doi:10.1186/s13613-024-01408-w)
Supplement: Supplementary file 1 — Supplementary Material 1 [file 13613_2024_1408_MOESM1_ESM.docx]

High flow nasal cannula and low level continuous positive airway pressure have different physiological effects during *de novo* acute hypoxemic respiratory failure

Samuel Tuffet^1,2,3^, MD ; Mohamed Ahmed Boujelben^2,3,4^, MD ; Anne-Fleur Haudebourg^2,3,4^, MD ; Tommaso Maraffi^2,3,5^, MD ; François Perier^6^, MD; Pascale Labedade^2,4^, MD ; Elsa Moncomble^2,3,4^, MD; Ségolène Gendreau^2,3,4^, MD; Matthieu Lacheny^4^; Emmanuel Vivier^1,2^, MD, PhD; Armand Mekontso-Dessap^2,3,4^, MD, PhD ; Guillaume Carteaux^2,3,4^, MD, PhD

Table of contents

[Supplementary methods 3](#_Toc172020397)

[Electrical impedance tomography 3](#_Toc172020398)

[Conversion between impedance and volume variation 3](#_Toc172020399)

[Estimation of tidal volume under HFNC 3](#_Toc172020400)

[End expiratory lung volume 3](#_Toc172020401)

[Global inhomogeneity index 4](#_Toc172020402)

[Distribution of ventilation 5](#_Toc172020403)

[Thoraco-abdominal asynchrony 5](#_Toc172020404)

[Supplementary results 7](#_Toc172020405)

[Figure S1. Individual patient representation of tidal volume variation between HFNC 1 and CPAP 7](#_Toc172020406)

[Figure S2. Spearman correlation between tidal volume during HFNC 1 and the variation in tidal volume between HFNC 1 and CPAP 8](#_Toc172020407)

[Table S1. Comparison of HFNC 1, CPAP and HFNC 2 in the 29 patients included 9](#_Toc172020408)

[Table S2. Comparison of tidal volume among patients subsequently intubated, versus not intubated 11](#_Toc172020409)

[Table S3. Comparison of HFNC 1 and CPAP in the 12 patients with arterial blood gas under CPAP 12](#_Toc172020410)

[Table S4. Comparison of patients according to the ROX score at inclusion 13](#_Toc172020411)

[References 15](#_Toc172020412)

# Supplementary methods

## Electrical impedance tomography

### Conversion between impedance and volume variation

The proportionality ratio between volume changes and electrical impedance changes was calculated using the CPAP period, as previously described (1): during the last minute of CPAP, the ratio k between tidal volume and tidal impedance change was calculated for each respiratory cycle. These ratios were averaged and used to convert the tidal impedance changes during HFNC to tidal volumes.

### Estimation of tidal volume under HFNC

The tidal volume (Vt) under HFNC was calculated from the tidal impedance changes under HFNC. As described previously (1), tidal volume was computed as

$$Vt= \Delta Ztidal x k$$

Where Vt is the tidal volume, ΔZtidal is the tidal impedance variation (on the global plethysmogram), and k the proportionality ratio between tidal volume and tidal impedance changes.

### Pendelluft

The Pendelluft phenomenon was computed as previously described (2,3). Tidal impedance was calculated for each pixel of the 32x32 matrix, defining the breath cycle for each pixel (Pixel-end-inspi Z_pixel_ – Pixel-end-exp Z_pixel_). This method considers the maximal ventilation of each pixel, independently of the global impedance variation. Tidal impedance variation at the pixel level (ΔZtidal _pixel-level_) was defined as the sum of the maximal impedance variation for every pixel. This parameter accounts for the Pendelluft phenomenon. Pendelluft was then calculated as:

$Pendelluft \left( \% \right)= \frac{{\Delta Ztidal}_{pixel-level}- \Delta Ztidal}{{\Delta Ztidal}_{pixel-level}}$ * 100

### End expiratory lung volume

The change in end-expiratory lung volume (EELV) between stages was calculated from the changes in end-expiratory lung impedance (EELI) between stages as follows

1. EELI was calculated for each step by averaging the end-expiratory impedance of each cycle during the last minute of each step.
2. Changes in end-expiratory impedance between each step were calculated, and converted to changes in end-expiratory volume by multiplying by the proportionality ratio k between changes in volume and changes in electrical impedance (1)
3. For better clarity, the end-expiratory impedance during HFNC 1 was taken as the reference, equal to 0. End-expiratory impedances during CPAP and HFNC 2 are thus expressed as difference from HFNC1.

Definition of the pixels involved in the ventilation

A ventilation map, representing the distribution of ventilation at the pixel level, was obtained for each condition using dedicated software (TIMPEL SA, São Paulo, Brazil). We determined for each map the pixel receiving the most volume (Pixel_max_). For each patient, among the three Pixel_max_ obtained (HFNC1, CPAP, HFNC2), the one receiving the lowest volume was taken as reference (Pixel_REF_). On each of the three maps corresponding to a patient, a pixel was considered ventilated if it received a volume greater than 10% of the volume received by the Pixel_REF_. This is an adaptation of an already widely published method (4,5), modified to account for tidal volume variations between different conditions. The changes in functional lung size between the three conditions were then calculated on the one hand in absolute value (change in the number of pixels, Δ Pixel_VENT, ABS_) and in a relative way, in order to index the functional size variation of the lung on the initial functional size (Δ Pixel_VENT, REL_ = Δ Pixel_VENT, ABS_ / Pixel_VENT_)

### Global inhomogeneity index

The Global inhomogeneilty index (GI) was calculated as described in (6) :

$$GI= \frac{\sum_{xy,lung} [\mathrm{DI}_{\mathrm{xy}}-Median\left( \mathrm{DI}_{\mathrm{lung}} \right)]}{\sum_{x,y,lung} \mathrm{DI}_{\mathrm{xy}}}$$

Where DI is the value of the differential impedance in the tidal images, DIxy is the pixel in the identified lung area, and DIlung is all the pixels representing the lung area. A pixel was considered to represent the lung if it was ventilated in at least one condition.

### Distribution of ventilation

The distribution of ventilation (anterior versus posterior) was computed based on the distribution of tidal impedance changes on the ventilation maps. Ventilation maps were divided horizontally in two equal layers (one anterior, one posterior). The percentage of anterior ventilation was computed as the sum of tidal impedance variation of anterior pixels, divided by the tidal impedance variation of the whole lung, multiplied by 100. The percentage of posterior ventilation was computed as the sum of tidal impedance variation of posterior pixels, divided by the tidal impedance variation of the whole lung, multiplied by 100.

In the main manuscript, only the anterior ventilation is indicated, the information on the percentage of anterior ventilation and the percentage of posterior ventilation being redundant. Indeed,

$$Anterior ventilation \left( \% \right)+Posterior ventilation \left( \% \right)=100$$

## Thoraco-abdominal asynchrony

During each stage (HFNC 1, CPAP, HFNC 2), thoracic and abdominal respiratory movements were recorded using two stretch-sensitive respiratory transducers (one around the chest, the other around the abdomen) (BioNomadix® Respiration Transducer, Biopac systems, Goleta, CA, USA), connected to a wireless transmitter (BioNomadix 2Ch Wireless RSP Transmitter, Biopac systems, Goleta, CA, USA).

The signals were then processed using AcqKnowledge version 4.3 (Biopac systems, Goleta, CA, USA).

The phase angle between thoracic and abdominal movements was calculated according to the method described by Agostoni (7). Thoracic and abdominal movements were plotted on a Lissajous figure (see figure S1). The direction of the diagram and the chronology (movement of the thorax before the abdomen, or of the abdomen before the thorax) made it possible to determine the phase angle between 0 and 360°, according to the equation (8) :

$$\sin\theta=\frac{m}{s}$$

Where m is the length of the midpoint of the rib cage excursion and s is the length depicting the abdominal excursion.

A phase angle equal to 0° or 360° corresponds to a perfect synchrony between the thorax and the abdomen. Conversely, a phase angle equal to 180° corresponds to a total asynchrony (paradoxical respiration). Thus, between 0° and 180°, the higher θ is, the greater the asynchrony. Conversely, between 180° and 360°, the higher θ is, the lower the asynchrony. In order to account for this bimodal relationship, we calculated a modified phase angle θ_mod_ according to:

- When 0° < θ < 180° : θ_mod_ = θ
- When 180° < θ < 360° : θ_mod_ = 360 – θ

Thus, regardless of θ, the higher θ_mod_ , the greater the thoracoabdominal asynchrony.

### Figure S1. Figure describing the phase angle calculation


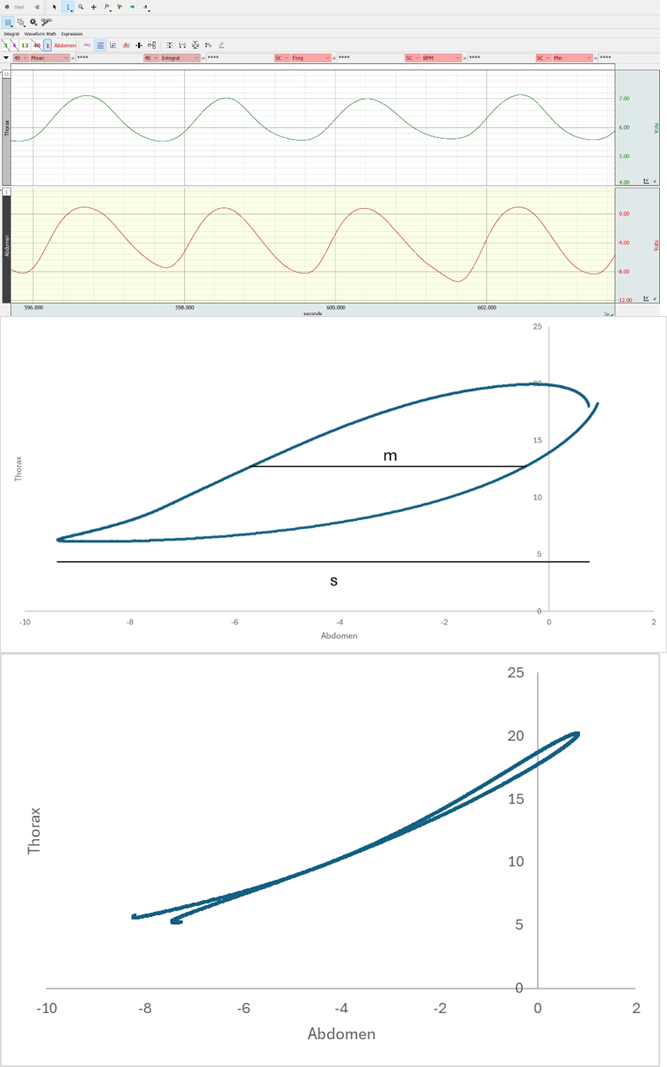


Top: Chest and abdominal movements as seen in AcqKnowledge software version 4.3 (Biopac systems, Goleta, CA, USA). Green: chest movements. Red: Abdomen movements. Of note, the scale of the y-axis is arbitrary.

Middle : typical Lissajous figure where chest movements are plotted against abdominal movements. The parameters m and s are shown. Phase angle is 31°.

Bottom : remarkable Lissajous figure in a patient with excellent synchrony between thoracic and abdominal movements

# Supplementary results

## Figure S2. Individual patient representation of tidal volume variation between HFNC 1 and CPAP


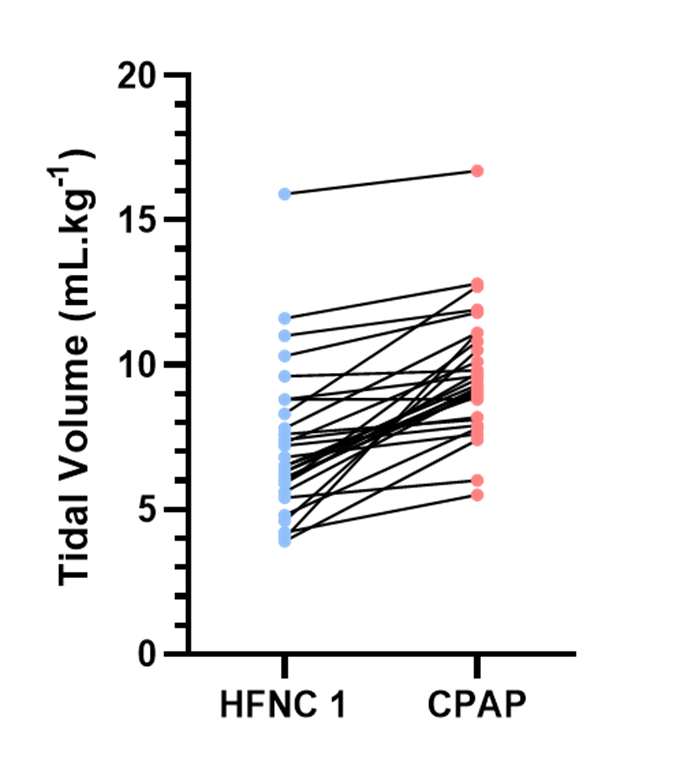


HFNC: High Flow Nasal Cannula; CPAP: Continuous Positive Airway Pressure

## Figure S3. Spearman correlation between tidal volume during HFNC 1 and the variation in tidal volume between HFNC 1 and CPAP


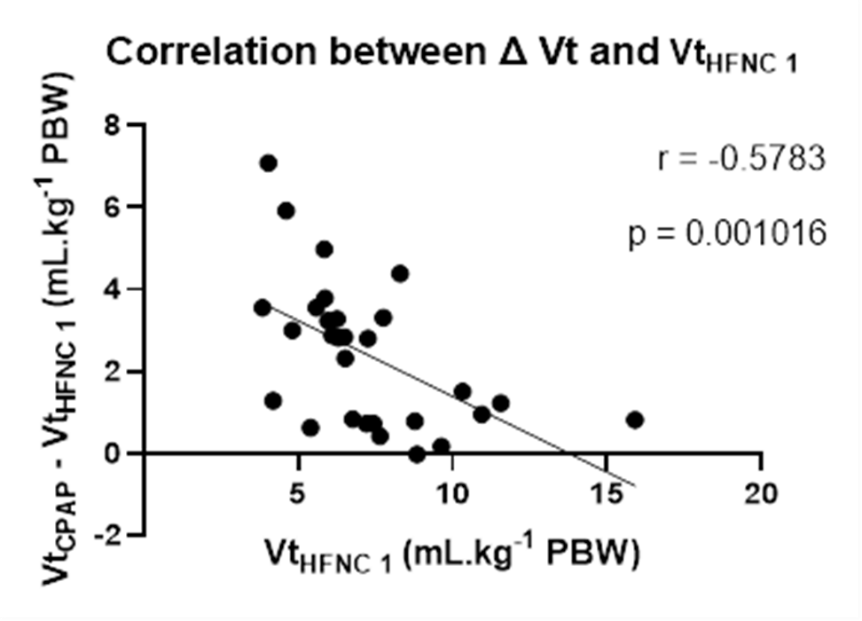


HFNC: High Flow Nasal Cannula; CPAP: Continuous Positive Airway Pressure

## Table S1. Comparison of HFNC 1, CPAP and HFNC 2 in the 29 patients included

| Variable | HFNC 1 | CPAP | HFNC 2 | p |
| --- | --- | --- | --- | --- |
| F_i_O_2_ (%) | 70 (50-100) | 70 (50-100) | 70 (50-100) | NA |
| Flow (L.min^-1^) | 50 (50-60) | NA | 50 (50-60) | NA |
| Respiratory rate (.min^-1^) | 24 (19-28) | 24 (20-30) | 25 (22-30) | 0.06 |
| Tidal volume (mL) | 453 (353-578) | 629 (551-686) * ° | 448 (378-576) | <0.01 |
| Tidal volume/PBW (mL.kg^-1^ PBW) | 6.6 (5.9-8.7) | 9.4 (8.3-11)*° | 6.9 (5.8-7.9) | <0.01 |
| Minute ventilation (L.min^-1^) | 10.6 (7.9-13.8) | 15.8 (12.5-17.5) *° | 11.2 (8.8-13.1) | <0.01 |
| EELV variation (mL) | 0 | 170 (43-399)*° | 46 (-16-170) | <0.01 |
| EELV variation/PBW (mL.kg^-1^ PBW) | 0 | 2.5 (0.6-5.4) *° | 0.6 (-0.3-2.5) | <0.01 |
| Thoraco-abdominal phase angle (°) (N=20) | 15 (9-34) | 10 (7-21) | 11 (9-32) | 0.43 |
| S_p_O_2_ (%) | 96 (95-99) | 99 (97-100)*° | 97 (95-99) | <0.01 |
| Dyspnea (n=23) | 0 | 0 (-1-1) | NA | 0.15 |
| Discomfort (n=23) | 0 | 1 (0-1) * | NA | 0.02 |
| ROX | 5.7 (4.2-8.2) | 5.5 (4.1-8.5) | 5.8 (4.3-8.6) | 0.25 |
| Anterior distribution (%) | 46 (41-54) | 41 (37-50)*° | 44 (42-52) | <0.01 |
| Global inhomogeneity index | 0.56 (0.53-0.60) | 0.58 (0.53-0.60) | 0.56 (0.53;0.59) | 0.87 |
| Ventilated pixels (n) | 327 (292-356) | 358 (341-394)*° | 334 (299-364) | <0.01 |
| Hemodynamic parameters | | | | |
| Heart rate (beat.min^-1^) | 87 (72-97) | 87 (71-103) | 86 (72-99) | 0.06 |
| SAP (mm Hg) | 123 (117-139) | 134 (115-146)* | 130 (118-145) | <0.01 |
| DAP (mm Hg) | 69 (57-75) | 74 (61-78)*° | 71 (57-76) | <0.01 |
| PP (mm Hg) | 55 (49-68) | 58 (48-77) | 61 (46-74) | 0.21 |
| Arterial blood gases (n=12) | | | | |
| P_a_O_2_/F_i_O_2_ (mm Hg) | 109 (77-160) | 153 (109-194)* | NA | <0.01 |
| P_a_CO_2_ (mm Hg) | 33 (31-35) | 35 (34-37)* | NA | <0.01 |
| pH | 7.48 (7.44-7.5) | 7.45 (7.41-7.48)* | NA | <0.01 |
| [HCO_3_^-^] (mmol.L^-1^) | 24.6 (24-26.5) | 25.6 (23.6-26.8) | NA | 0.11 |
| Corrected minute ventilation (L.min^-1^) | 8.5 (7.4-11.3) | 14.6 (11.4-15.8)* | NA | <0.01 |
| Ventilatory ratio | 1.39 (1.11-1.67) | 2.15 (1.91-2.6) | NA | 0.001 |

End-expiratory lung volumes during CPAP and HFNC 2 are expressed as difference from HFNC1.

HFNC : High Flow Nasal Cannula ; CPAP : Continuous Positive Airway Pressure ; F_i_O_2_ : inspired fraction of oxygen ; PBW : predicted body weight ; EELV : end-expiratory lung volume ; S_p_O_2_ : oxygen saturation by pulse oximeter ; SAP : systolic arterial pressure ; DAP : diastolic arterial pressure ; PP : pulse pressure ; P_a_O_2_ : arterial oxygen pressure ; F_i_O_2_ : inspired fraction of oxygen ; P_a_CO_2_ : arterial carbon dioxide pressure ; last column indicates either the result of the Friedman test or the result of the Wilcoxon paired test, as appropriate; * denotes p<0.05 versus HFNC 1 ; ° denotes p< 0.05 versus HFNC 2

## Table S2. Comparison of tidal volume among patients subsequently intubated, versus not intubated

|  | No Intubation  (n=18) | Intubation  (n=11) | p-value |
| --- | --- | --- | --- |
| Tidal volume during HFNC 1 (mL.kg^-1^ PBW) | 6.1 (5.2-8.1) | 7.2 (6.3-8.8) | 0.17 |
| Tidal volume during CPAP (mL.kg^-1^ PBW) | 9.5 (8.7-11.1) | 9.3 (7.9-9.8) | 0.72 |

HFNC: High Flow Nasal Cannula; CPAP: Continuous Positive Airway Pressure; PBW: predicted body weight

## Table S3. Comparison of HFNC 1 and CPAP in the 12 patients with arterial blood gas under CPAP

| Variable | HFNC 1 | CPAP | p |
| --- | --- | --- | --- |
| F_i_O_2_ (%) | 82 (63-100) | 82 (63-100) | NA |
| Flow (L.min^-1^) | 60 (50-60) | NA | NA |
| Respiratory rate (.min^-1^) | 25 (20-28) | 25 (21-30) | 0.38 |
| Tidal volume (mL) | 423 (326-578) | 647 (571-688) | <0.001 |
| Tidal volume (mL.kg^-1^ PBW) | 6.4 (5.1-8.1) | 9.4 (7.9-11) | <0.001 |
| Minute ventilation (L.min^-1^) | 10.3 (8-14.6) | 16.5 (13.1-18) | 0.002 |
| EELV (mL) | 0 | 199 (63-390) | 0.001 |
| EELV/PBW (mL.kg^-1^ PBW) | 0 | 2.6 (0.9-6.3) | 0.001 |
| SpO_2_ (%) | 96 (95-99) | 99 (97-99) | 0.02 |
| ROX (%.min) | 5.1 (4.2-7.3) | 4.8 (3.8-7.3) | 0.87 |
| Anterior distribution (%) | 43 (41-56) | 39 (36-49) | 0.03 |
| Global inhomogeneity index | 0.58 (0.55-0.61) | 0.58 (0.54-0.61) | >0.99 |
| Ventilated pixels (n) | 301 (284-346) | 355 (317-392) | <0.001 |
| Heart rate (.min^-1^) | 89 (68-103) | 89 (68-103) | 0.98 |
| SAP (mmHg) | 129 (119-144) | 139 (132-148) | 0.01 |
| DAP (mmHg) | 61 (56-74) | 66 (59-78) | 0.002 |
| PP (mmHg) | 64 (55-73) | 69 (54-80) | 0.06 |
| P_a_O_2_/F_i_O_2_ (mmHg) | 109 (77-160) | 153 (109-194) | 0.004 |
| P_A_CO_2_ (mmHg) | 33 (31-35) | 35 (34-37) | <0.001 |
| pH | 7.48 (7.44-7.5) | 7.45 (7.41-7.48) | <0.01 |
| HCO_3_- | 24.6 (24-26.5) | 25.6 (23.6-26.8) | 0.11 |
|  |  |  |  |

HFNC : High Flow Nasal Cannula ; CPAP : Continuous Positive Airway Pressure ; F_i_O_2_ : inspired fraction of oxygen ; PBW : predicted body weight ; EELV : end-expiratory lung volume ; S_p_O_2_ : oxygen saturation by pulse oximeter ; SAP : systolic arterial pressure ; DAP : diastolic arterial pressure ; PP : pulse pressure ; P_a_O_2_ : arterial oxygen pressure ; F_i_O_2_ : inspired fraction of oxygen ; P_a_CO_2_ : arterial carbon dioxide pressure ;

## Table S4. Comparison of patients according to the ROX score at inclusion

|  | ROX < 5.7  N=15 | ROX > 5.7  N=14 | p |
| --- | --- | --- | --- |
| Data under HFNC 1 | | | |
| F_i_O_2_ (%) | 100 (80-100) | 50 (45-60) | <0.01 |
| Flow (L.min^-1^) | 60 (50-60) | 50 (50-56) | 0.18 |
| Respiratory Rate (breaths.min^-1^) | 26 (21-30) | 21 (17-25) | 0.02 |
| Tidal volume (mL.kg^-1^ PBW) | 6.8 (5.9-7.8) | 6.3 (5.2-10.5) | 0.96 |
| Minute ventilation (L.min^-1^) | 10.7 (8.9-14.8) | 9.9 (7.6-10.9) | 0.39 |
| Thoraco-abdominal phase angle (°) (N=20) | 24 (9-40) | 13 (7-31) | 0.28 |
| SpO2 (%) | 96 (95-99) | 98 (95-99) | 0.53 |
| Dyspnea (n=23) | n=12  5 (1-6) | n=11  5 (0-6) | 0.51 |
| Discomfort (n=23) | n=12  5 (1-6) | n=11  4 (0-5) | 0.2 |
| ROX | 4.3 (3.4-5.3) | 8.2 (7.5-12.1) | <0.01 |
| Anterior distribution (%) | 44 (41-54) | 48 (42-55) | 0.39 |
| Global inhomogeneity index | 0.59 (0.55-0.61) | 0.54 (0.49-0.56) | <0.01 |
| Number of ventilated pixels | 294 (255-338) | 350 (319-368) | <0.01 |
| Heart rate (.min^-1^) | 89 (71-112) | 80 (73-91) | 0.29 |
| SAP (mm Hg) | 127 (119-139) | 121 (115-137) | 0.42 |
| DAP (mm Hg) | 65 (56-75) | 69 (56-75) | 0.93 |
| PP (mm Hg) | 62 (52-68) | 55 (46-69) | 0.41 |
| P_a_O_2_/F_i_O_2_ (mm Hg) | 109 (86-128) | 159 (115-201) | 0.05 |
| P_a_CO_2_ (mm Hg) | 33 (32-35) | 34 (28-43) | 0.68 |
| Variations between HFNC 1 and CPAP (CPAP minus HFNC1) | | | |
| Δ Respiratory Rate (.min^-1^) | 3 (0-6) | 1 (-2-4) | 0.20 |
| Δ Tidal volume (mL.kg^-1^ PBW) | 2.8 (0.8-3.6) | 1.4 (0.8-3.3) | 0.4 |
| Δ Minute ventilation (L.min^-1^) | 6 (3.8-6.7) | 3.5 (1-6) | 0.03 |
| Δ EELV (mL.kg^-1^ PBW) | 2.3 (0.6-5.5) | 2.5 (-0.3-5.7) | 0.75 |
| Δ Thoraco-abdominal phase angle (n=20) | N=10  -9 (-21-12) | N=10  -4 (-14-0) | 0.88 |
| Δ SpO_2_ (%) | 2 (1-3) | 1 (0-3) | 0.34 |
| Δ Dyspnea (Likert) (n=23) | N=12  0 (-1-2) | N=11  0 (-1-1) | 0.59 |
| Δ Discomfort (Likert) (n=23) | 1 (-1-1) | 1 (0-2) | 0.75 |
| Δ anterior distribution (%) | -2 (-6-1) | -4 (-7-0) | 0.57 |
| Δ GI | 0 (-0.04-0.02) | 0.02 (-0.01-0.04) | 0.06 |
| Δ Pixel_VENT, ABS_ (n) | 47 (30-64) | 29 (12-53) | 0.06 |
| Δ Pixel_VENT, REL_ (%) | 16 (10-26) | 9 (3-14) | 0.02 |
| Δ Heart rate (.min^-1^) | 1 (-2-5) | 2 (0-5) | 1 |
| Δ SAP (mmHg) | 7 (4-14) | 5 (-1-8) | 0.1 |
| Δ DAP (mmHg) | 4 (2-7) | 3 (-1-6) | 0.21 |
| Δ PP (mmHg) | 4 (-2-8) | 1 (-3-6) | 0.33 |
| Δ P_a_O_2_/F_i_O_2_ (mmHg) | n=9  58 (10-67) | n=3  21 (11-124) | 0.85 |
| Δ P_a_CO_2_ (mmHg) | n=9  3 (2-4) | n=3  2 (1-2) | 0.19 |

F_i_O_2:_ inspired fraction of oxygen; PBW : predicted body weight; S_p_O_2_: oxygen saturation by pulse oximeter; P_a_O_2_: arterial oxygen pressure; P_a_CO_2_ : arterial carbon dioxide pressure; EELV: end-expiratory lung volume; Δ Pixel_VENT, ABS_: absolute variation in the number of ventilated pixels between HFNC 1 and CPAP; Δ Pixel_VENT, REL_: relative variation in the number of ventilated pixels between HFNC 1 and CPAP.

Patients were separated into two groups according to the median ROX index (9) at inclusion to determine the differential effects of CPAP in the most severe patients. Patients at higher risk for intubation (ROX score > 5.7) were older [65 years (61-74) versus 59 years (33-67), p=0.04], had higher BMI [26.6 kg.m^-2^(20.7-31.1) versus 21.7 kg.m^-2^(19.05-24.1), p=0.04], and a lower P_a_O_2_/F_i_O_2_ ratios [109 mmHg (86-128) versus 159 mmHg (115-201), p=0.05]. During HFNC 1, these patients had greater ventilation inhomogeneity and smaller functional lung size (Table 3). Among patients with ROX < 5.7, CPAP was characterized by a similar increase in tidal volume and EELV but a greater increase in minute ventilation and a greater increase in relative functional lung size (Table 3). The ROX and the tidal volume variation between HFNC 1 and CPAP were not significantly correlated.

# References

1. Mauri T, Turrini C, Eronia N, Grasselli G, Volta CA, Bellani G, et al. Physiologic Effects of High-Flow Nasal Cannula in Acute Hypoxemic Respiratory Failure. Am J Respir Crit Care Med. 2017 01;195(9):1207–15.

2. Menga LS, Delle Cese L, Rosà T, Cesarano M, Scarascia R, Michi T, et al. Respective Effects of Helmet Pressure Support, Continuous Positive Airway Pressure, and Nasal High-Flow in Hypoxemic Respiratory Failure: A Randomized Crossover Clinical Trial. Am J Respir Crit Care Med. 2023 May 15;207(10):1310–23.

3. Grieco DL, Delle Cese L, Menga LS, Rosà T, Michi T, Lombardi G, et al. Physiological effects of awake prone position in acute hypoxemic respiratory failure. Crit Care. 2023 Aug 17;27(1):315.

4. Spinelli E, Kircher M, Stender B, Ottaviani I, Basile MC, Marongiu I, et al. Unmatched ventilation and perfusion measured by electrical impedance tomography predicts the outcome of ARDS. Crit Care. 2021 Jun 3;25(1):192.

5. Pavlovsky B, Pesenti A, Spinelli E, Scaramuzzo G, Marongiu I, Tagliabue P, et al. Effects of PEEP on regional ventilation-perfusion mismatch in the acute respiratory distress syndrome. Crit Care. 2022 Jul 11;26(1):211.

6. Zhao Z, Möller K, Steinmann D, Frerichs I, Guttmann J. Evaluation of an electrical impedance tomography-based global inhomogeneity index for pulmonary ventilation distribution. Intensive Care Med. 2009 Aug 4;35(11):1900.

7. Agostoni E, Mognoni P. Deformation of the chest wall during breathing efforts. J Appl Physiol. 1966 Nov;21(6):1827–32.

8. Hammer J, Newth CJL. Assessment of thoraco-abdominal asynchrony. Paediatr Respir Rev. 2009 Jun 1;10(2):75–80.

9. Roca O, Caralt B, Messika J, Samper M, Sztrymf B, Hernández G, et al. An Index Combining Respiratory Rate and Oxygenation to Predict Outcome of Nasal High-Flow Therapy. Am J Respir Crit Care Med. 2019 Jun;199(11):1368–76.
